# Distinctions and Affinities Between Leadership Emergence and Leadership Effectiveness

**DOI:** 10.3389/fpsyg.2021.624377

**Published:** 2021-08-12

**Authors:** Micha Popper

**Affiliations:** Department of Psychology, University of Haifa, Haifa, Israel

**Keywords:** leadership emergence, leadership effectiveness, followership, attribution, projection

There is a growing understanding that the foundations of followership are the key to deciphering the nature of leadership. (Shamir et al., 2007; Lapierre and Carston, 2014; Popper, 2014; Uhl-Bien et al., 2014; Popper and Castelnovo, 2018).

This claim is central in the article's attempt to understand processes underlying leaders' emergence and the evaluation of leaders' effectiveness.

Two questions are addressed prior to presenting propositions that deal with the distinctions and affinities of both notions.

(A) Why are leaders so important to followers?

(B) How do the factors that determine leaders' centrality for followers affect their reference to leadership effectiveness?

## Why Are Leaders so Important to Followers?

Three major explanations are suggested to explain the importance of leaders to followers: (1) the evolutionary explanation; (2) the psychodynamic explanation; and (3) the social-psychological explanation.

### The Evolutionary Explanation

According to the *evolutionary perspective*, most species are equipped with an innate, pre-learned tendency to be attracted to figures sensed as being “wise and strong” (Mayseless and Popper, 2019). Evolutionarily, this tendency enables the preservation of the human infant's survival during the prolonged period in which he is totally dependent on other care-taking figures. (Bowlby, 1973). The relationship between the “small figure” (who needs protection and adaptive knowledge) and the “large figure” (e.g., a care-giver in infancy and various authority figures later) is partly accomplished through informative signals (Antonakis et al., 2016). For example, among animals, certain signs express power which may lend their subjects leadership status. This is also the case with humans—with one crucial difference: humans are uniquely sensitive to symbolic signals, such as language (Csibra and Gergely, 2006, 2009; Tomasello, 2014).

The important evolutionary signals of leadership stem from two complementary groups: signals of competence and signals of care (Castelnovo et al., 2017); meaning, signals sensed as reflecting the ability to deal with adaptive challenges (Todorov et al., 2005; van Vugt et al., 2008; van Vugt and Grabo, 2015; Grabo et al., 2017), along with the sense that this competence is aimed at the followers' needs (Fiske et al., 2007; Csibra and Gergely, 2009). For example, the leader in ancient times was not only sensed as one who knew where to find water, but also as someone who would make sure the water was shared (Boehm, 1999). These two phylogenetic signals are major antecedents of leadership emergence (Grabo et al., 2017; Popper and Castelnovo, 2018).

### The Psychodynamic Explanation

According to the psychodynamic explanation, the sources of attraction to a leader are unconsciously formed in early childhood during the period of total impotence. Hence, the yearning for a leader is simply a longing for parental figures who can provide us with protection and care (Freud, 1939; Bowlby, 1973; Hill, 1984).

According to such explanations, leadership is a *projection*—if the “appropriate signals” of strength and care are sensed as being associated with a certain figure, s/he will be accepted as a leader—as the right response to followers' anxieties and desires.

### The Social-Psychological Explanation

The reality in which people live is also replete with meanings represented by symbols (Charon, 1979). Leaders in this context can also be symbolic representations of a cultural and/or social category that assist in defining the followers' social self (Shamir et al., 1993; Hofstede, 1997; Allison and Goethals, 2011). Leaders are a sort of narrative through which group identity can be crystalized (e.g., Shamir et al., 1993; Hogg, 2001).

Some scholars claim. that humans' advantages in the face of adaptive challenges stem from the ability to work in a group. The more coordinated group functions were, the more effective the group was (Tomasello, 2014; van Vugt and Grabo, 2015). Leaders were those individuals who coordinated the group's activities when facing challenges (van Vugt and Grabo, 2015). Arguably, with the advancement of symbolic forms, leaders, through symbolic means, were able to enhance functions that had existed in less sophisticated forms at the beginning of human evolution (Harari, 2015; Popper and Castelnovo, 2018).

The conceptual framework presented thus far allows us to analyze the impact of the most significant factors that affect leadership emergence.

### The Feasibility and Intensity of Leadership Emergence

As can be seen from the above explanations, there is a conceptual hierarchy underpinning the yearning for a leader. The most fundamental, is the evolutionary explanation, which is at the base of the inherent longing for competent, and caring figures alongside the constant seeking of mechanisms that preserve the collective entity. This understanding sheds light on key aspects that accelerate leadership emergence. The most prominent among them are discussed below.

#### Psychological Distance

From the followers' perspective, two psychological phenomena, which are unique to humans, serve to intensify the longing for leaders. One, as mentioned, is the human ability to *project*, which during times of crisis magnifies leadership (e.g., Popper, 2001; Volkan, 2004; Lipman-Blumen, 2007). The other is humans' typical *attribution patterns*. Both phenomena are largely affected by psychological distance.

*Psychological distance* is defined by Liberman et al. (2007) as a mental construal: **“…**on a single starting point (zero distance point) which is a *direct experience* of the here and now. Anything else—other times, other places, experiences of other people, and hypothetical alternatives to reality, is a mental construal” (p. 353).

Construal Level Theory (CLT) (Liberman and Trope, 1998) suggests two levels of construal and proposes that more distal entities are construed on a higher level, that is, involve more construal. The reason for this is that as we move away from our direct experience of things, we have less information about those things.

Abstract representations are simpler and more prototypical than concrete representations (Fiske and Taylor, 1991; Smith, 1998). In feature-based theories of categorization, more inclusive categories have fewer features and are therefore simpler than concrete categories (Rosch and Lloyd, 1978). In the same way, abstract traits are less detailed about the behaviors and circumstances, they involve (Hampson et al., 1986).

This argument was reflected in Shamir's study (Shamir, 1995). The participants were asked to describe two types of leaders: a *close leader* with whom they had direct contact, and a *distant leader* with whom they had never had direct contact. A comparative analysis of the frequency and contents of the adjectives used to describe the two types of leaders revealed that distant leaders were described by more general traits, and were characterized by fewer adjectives and less daily behaviors than close leaders.

The relevance of the arguments and research on psychological distance with regard to the emergence of leaders is clear. The ability of followers to validate leadership signals in close leaders is greater, as it is easier to attribute causality to close leaders when there are many observed behaviors and concrete outcomes (Erickson and Krull, 1999). Hence, the distance from the leader does not necessarily lead to a valid completion of the interpretation known in the literature as *correspondence inference* (Erickson and Krull, 1999). As a result, there are more perceptual limitations with regard to the congruence between behaviors, traits, and outcomes in terms of attribution theories (Hamilton, 1988; Lopez and Ensari, 2014). Hence, the options for manipulating the choice of “false leaders” are more feasible in the context of distant leaders.

#### Cultural Differences

Studies in the context of leadership and culture indicate differences in leadership imagery among different cultures (Gestner and Day, 1994; Dorfman, 1996; Hofstede, 1997; Den Hartog et al., 1999). For example, Gestner and Day, 1994 compared leadership perceptions among students in eight countries. They presented the subjects with 59 leadership characteristics and found that their level of agreement was small.

## Uncertainty

Uncertain situations intensify the inherent yearning for a “large, competent figure.” The more acute one's sense of uncertainty is, the more intensive his/her yearning for a (strong) leader to emerge, will be (Hertzler, 1940; Pillai, 1996; Popper, 2001).

To summarize this section, it is argued that leadership emerges in different magnitudes and ways; at different distances from the leader (Shamir, 1995; Popper, 2013); according to the weight the culture ascribes to authority figures (Hofstede, 1997; Den Hartog et al., 1999); and at different levels of change, and crisis (van Vugt and Grabo, 2015; Castelnovo et al., 2017). The common denominator of all the discussed claims is that leadership emergence is, in many circumstances, an emotionally-biased phenomenon (Popper and Castelnovo, 2018).

Following this premise, we suggest several propositions that may illuminate the distinctions as well as the affinities existing between leadership emergence and leadership effectiveness.

### Proposition 1-The Central Heuristics Used by Followers to Assess Leadership Effectiveness Are Based on Results

In a study conducted by Lipshitz (1991), officers evaluated four versions of the same decision-making case. In two versions, the decision-maker obeys orders, while in the other two versions he disobeys them. One out of each decision is successful; one out of each decision fails. Although the cases are identical successful decision-makers are perceived more favorably than their unsuccessful counterparts. The study shows that successful outcomes overshadow the decision-making process, even if the success was reached by contradicting the rules or ignoring important data. This link between success and leadership has been explained as an expression of two cognitive biases:

1. *Fundamental attribution error*—the tendency to ascribe greater weight to “actors” than to circumstances (Ross et al., 1977). Leadership is a clear manifestation of this bias. Success is often overly attributed to leaders. For example, Meindl et al. (1985) provided participants with vignettes of organizational events, and measured the extent to which participants attributed the event either to leaders or to other causes. All other causes were equally likely and plausible, but the results indicated that participants were more inclined to explain the event in terms of the leader compared to all other explanations.
2. *Availability bias*—the general tendency to assess events by the ease with which occurrences come to mind (Kahneman, 2011). Leaders provide particularly “salient and accessible information” (Popper, 2012, p. 32), when it comes to evaluating informatively complex situations (Lord et al., 1984). Such biases lead to overemphasizing leaders' contributions to successful organizational results. Moreover, sometimes successful outcomes glorify a person who happened to be in in a formal leadership position, even though the outcomes did not necessarily stem from his/her decisions, (e, Spector, 2014)

These general heuristics linking leadership to results vary due to the factors discussed above.

### Proposition 2-Leadership Effectiveness and Distance

The closer the leader is, the more the effectiveness of his/her leadership will be assessed on the basis of the actual results of his/her organizational unit. The farther away the leader is, the more the assessment of his/her effectiveness will be based on generalized and abstract informative categories (“strong,” “determined”,) (Shamir, 1995; Liberman and Trope, 1998; Liberman et al., 2007; Popper, 2013).

The following propositions are anchored in a different paradigm according to which the reference toward leadership is largely inherent in cultural attributes or primordial elements that create different categories of evaluating leadership effectiveness.

### Proposition 3-The More Culturally Prototypical the Leader is Perceived in a Given Group, the More s/he Will Be Attributed With Effectiveness

The common argument suggested in many works (e.g., Gestner and Day, 1994; Dorfman, 1996; Hofstede, 1997; Den Hartog et al., 1999; Popper and Khatib, 2001) is that intercultural differences affect both: a) the centrality ascribed to a leader. b) the type of outcomes linked to the leader. For example, in collectivistic cultures, there is less inclination to attribute organizational effectiveness to leaders compared to individualistic societies. The success of organizations like Toyota is more attributed to variables such as the system's design and work methods (Liker, 2004). In contrast, the success of organizations in the US (an individualistic culture), such as General Electric and Chrysler, was attributed more to individual leaders (Popper, 2012; Spector, 2014).

### Proposition 4-In Stressful Situations the Leader's Effectiveness Will Be Determined by Feelings of Security Sensed by the Followers as Radiating From the Leader's Speeches and Behavior

This proposition is anchored in works conducted on leadership during times of crisis and change (Hertzler, 1940; Conger and Kanungo, 1987; Pillai, 1996; Popper, 2012). Common to all these studies is the conclusion that crisis situations highlight primary emotions (e.g., Kets de Vries, 1988) in relation to leaders. The effectiveness of leaders in such circumstances is essentially affected by the degree of confidence experienced by the followers from the leaders' public appearances. (Burns, 2002).

The main indicator of a leader's effectiveness in these situations is a sense of anxiety reduction (Kets de Vries, 1988), which often has nothing to do with the end results of the crisis (e.g., Lindholm, 1990; Kershaw, 1998, 2001).

In conclusion, it is argued that the evaluation of leadership effectiveness is affected by consequential thinking and is largely biased by outcomes, whereas leadership emergence is grounded in cultural biases and primordial feelings and is not necessarily associated with evaluable parameters. In this sense, although, as discussed, antecedents of leadership emergence can influence the evaluation of leadership effectiveness. Thus, we should keep in mind that the very essence of leadership emergence goes beyond common categories such as “successful”/“unsuccessful.”

## Author Contributions

The author confirms being the sole contributor of this work and has approved it for publication.

## Conflict of Interest

The author declares that the research was conducted in the absence of any commercial or financial relationships that could be construed as a potential conflict of interest.

## Publisher's Note

All claims expressed in this article are solely those of the authors and do not necessarily represent those of their affiliated organizations, or those of the publisher, the editors and the reviewers. Any product that may be evaluated in this article, or claim that may be made by its manufacturer, is not guaranteed or endorsed by the publisher.
